# ImproviNg Tic services in EnglaND: a multi-method study to explore existing healthcare service provision for children and young people with tics and Tourette syndrome

**DOI:** 10.1136/bmjment-2025-301599

**Published:** 2025-07-07

**Authors:** Nikita R Rattu, Sophie S Hall, Charlotte L Hall, Tara Murphy, Joseph Kilgariff, Nadya James, Emma McNally, Alexia Jeayes, Kareem Khan, Suzanne Rimmer, Louise Thomson, Madeleine Jane Groom

**Affiliations:** 1 Academic Unit of Mental Health & Clinical Neurosciences, University of Nottingham School of Medicine, Nottingham, UK; 2 Nottinghamshire Healthcare NHS Foundation Trust, Nottingham, UK; 3 School of Medicine, Nottingham Clinical Trials Unit, Nottingham, UK; 4 NIHR MindTech MedTech Health Research Centre, Institute of Mental Health, Nottingham, UK; 5 Royal Free Campus, Research Department of Primary Care and Population Health and Priment CTU, University College London, London, UK; 6 Great Ormond Street Hospital for Children NHS Foundation Trust, London, UK; 7 Nottingham University Hospitals NHS Trust, Nottingham, UK; 8 Tourettes Action, Farnborough, UK; 9 Former NHS Healthcare Commissioner (retired), Merseyside, UK

**Keywords:** child & adolescent psychiatry

## Abstract

**Background:**

Timely access to diagnostic assessment and treatment is essential to improve function and mitigate the risk of poor long-term outcomes in children and young people (CYP) with tics.

**Objective:**

This study aimed to explore (i) how tic services for CYP in England are currently organised, including access to assessment and treatment and (ii) healthcare professionals’ (HCPs) experiences of assessing and treating tics.

**Methods:**

Two methodologies were used to examine tic service provision. First, two freedom of information (FOI) requests were sent to Integrated Care Boards (FOI1) and service providers (FOI2) to gather data on referral and assessment processes, and treatments offered. Second, a national survey of HCPs explored their experiences and training needs when assessing and treating tics.

**Findings:**

FOI responses indicated that 12 of 62 services (19.4%), primarily located in the London area, offered a full pathway for the referral, assessment and treatment of tics in CYP.

The national survey sample (n=184) included psychologists, paediatricians, neurologists and mental health nurses. Most described services as poorly structured and reported a need for additional resources and training in the assessment and treatment of tics.

**Conclusions:**

Inconsistent and underfunded tic service provision across England limits HCPs’ ability to support CYP with tics effectively. There is an urgent need to develop clear service pathways offering both assessment and treatment, and to equip HCPs with sufficient training and resources to provide appropriate care.

**Clinical implications:**

Current tic service provision does not meet the healthcare needs of CYP in England. Without improvements, CYP are at increased risk of poorer long-term outcomes.

**WHAT IS ALREADY KNOWN ON THIS TOPIC**

- Tic disorders in children and young people are associated with poor mental health outcomes including increased risk of suicidality in adulthood.
- Healthcare services for this population have not been systematically evaluated in England, and there is limited prior research exploring the knowledge and experience of healthcare professionals (HCPs) in this area.

**WHAT THIS STUDY ADDS**

- Of the limited number of service providers in England with a full pathway for the assessment and treatment of tics, these are predominantly concentrated in London.
- Some HCPs have low confidence when assessing and treating tics and often have not received training to do so.

**HOW THIS STUDY MIGHT AFFECT RESEARCH, PRACTICE OR POLICY**

- The urgent need to improve tic services is clear, including appropriate allocation of funding, development of training resources for HCPs and consistent provision that includes both assessment and treatment.

## Background

 Tic disorders, including Tourette syndrome (TS), chronic motor or vocal tic disorder and provisional tic disorder, affect around 1% of children and young people (CYP).^1^ Tics are involuntary, repeated movements or vocalisations that can range from simple tics, such as eye blinking or throat clearing, to more complex behaviours involving coordinated actions or vocal expressions.

Without appropriate intervention, CYP are more likely to experience physical injuries from tics,^2^ poor mental health and low self-esteem.^3^ Most CYP with a tic disorder also experience co-occurring neurodevelopmental or psychiatric conditions that can exacerbate tics and require careful management.^3^ Comprehensive, ongoing care to identify and manage tics, alongside any co-occurring mental health and neurodevelopmental conditions, is essential to mitigate these risks and improve outcomes. Unmanaged tics can increase the risk of significant psychological and social challenges in adulthood, including mental health difficulties and suicidality^4^ and reduced quality of life.^5^ Poorer educational outcomes have also been identified in young people with tic disorders, even when controlling for the presence of other neurodevelopmental conditions.^6^ These outcomes have a wider, long-lasting negative impact on young people, contributing to poorer social and economic status.^7^ Interactions with other mental health and neurodevelopmental symptoms further increase the likelihood of poor outcomes, highlighting the need for assessment and management of tics and co-occurring conditions.^1 3^

Despite the recognised impact of tic disorders, CYP and their families describe significant challenges accessing healthcare services for tics in England. For example, in a survey of 1508 people with chronic tic disorders conducted by the National Institute for Health and Care Excellence (NICE) in 2024,^8^ 64.1% had waited over a year for diagnostic assessment, while >23% waited longer than 3 years. More than 61% of those given a diagnosis were not offered treatment. A systematic review^9^ and qualitative research^10^ show that barriers to accessing appropriate care include unclear and lengthy referral pathways with an average time of 3 years from tic onset to receiving support, a lack of locally available healthcare professionals (HCPs) with relevant training and knowledge, long waitlists and limited access to intervention following diagnosis. Evidence from case-study data suggests that inefficient referral pathways for CYP with tics, which often include multiple declined and repeat referrals, may cost the NHS around £3512.55 per person, whereas a streamlined, efficient process for referral from primary to specialist services would reduce the cost to around £1146.76 per person.^11^

Another significant challenge for people with tics is accessing appropriate treatment. Evidence-based treatment varies and may include behavioural therapies, such as comprehensive behavioural intervention for tics (CBIT) or exposure with response prevention (ERP),^12^ and pharmacological options.^9 13^ Specialist training is required to deliver behavioural therapy, and this, combined with limited capacity within community specialist services, means that evidence-based interventions are difficult to access, with previous research finding that only 19% of CYP in the UK were able to access treatment.^14^ Indeed, at a global, national, regional and local level, there is a lack of specialist tic services.^15 16^ It is important we understand the current structure of tic services for CYP in England to identify regional disparities and where improvements can be made. Existing research has surveyed HCPs in the UK on regional tic service provision^16^ and explored barriers and facilitators to providing effective healthcare to CYP with tics.^17^ However, there is currently no research that has explored in depth the experiences, confidence and training needs of HCPs in England when assessing and treating tics.

The current study aimed to assess how services for tic disorders are currently organised in England, and the experiences and training needs of HCPs.

## Methods

Two methodologies were used to gather information about tic service provision for CYP in England. First, freedom of information (FOI) requests were sent to healthcare providers in England to assess how services for tic disorders are currently organised. This included collecting information on referral processes, diagnostic practices and treatment options. Second, a national survey was disseminated to HCPs, to understand their experiences of assessing and treating tics in CYP, explore barriers and facilitators to providing effective care and identify training needs.

### Freedom of information requests

In the UK, the Freedom of Information Act (2000) requires public organisations to provide a response to specific questions issued by an individual or organisation within 20 working days. Using this approach in research increases response rates and provides comprehensive information.^18^

FOI requests were sent in two stages. The first request (FOI1) was sent to Integrated Care Boards (ICBs) in England to identify the named provider of tic services for CYP within that ICB. ICBs are statutory NHS organisations that manage and allocate resources to support healthcare in their defined area. Each ICB comprises one or more ‘places’, which refer to a local area within an ICB where health and care services are coordinated more closely to meet the needs of that specific population.

FOI1 was sent to each ICB, asking them to state the named service provider of tic services for every place within their ICB. The FOI request asked (1) for the contact details of tic service providers, (2) if places had plans to improve tic service provision and (3) if they were interested in clinical training and a best practice model. FOI1 was pilot-tested in eight ICBs in late July 2023, with minor changes made to improve item comprehension. FOI1 was sent electronically to all 42 ICBs, covering 295 places. ICBs were instructed to complete a spreadsheet with specific headings on behalf of each place. FOI1 requests were sent between 1 August 2023 and 5 August 2023, with responses received between 7 August 2023 and 6 September 2023.

#### Freedom of information second request

Once tic service arrangements for each ICB had been established through FOI1, including the named service provider (where one existed), a second FOI request (FOI2) was sent to all those named contacts to gather more detailed information about the tic service they provide.

This included information on the age range of patients accepted into the service, how referrals for tics were managed, the number of referrals received and accepted by the service and how many CYP with tics received diagnostic assessments and treatment. Services were asked to provide data for the period between 1 April 2022 and 31 March 2023.

FOI2 was pilot-tested by sending it to six service providers named in FOI1. Pilot FOI2 requests were sent on 30 January 2024. Responses were received from 21 February to 28 February 2024. Following minor adjustments to the wording of some questions, FOI2 requests were sent in March 2024 to all service providers named by ICBs in FOI1. Overall, FOI2 was sent to 80 service providers, covering 180 places, who provide a service for CYP with tic disorders. Most service providers were NHS trusts (n=55, 68.8%). Most responses were received between April and May 2024.

Both FOI1 and FOI2 were developed iteratively with input from clinical and research experts alongside the research team.

#### Analysis

Descriptive statistics were used to summarise responses to each of the questions contained within the FOI requests. Results were also reported separately by region.

### National survey of healthcare professionals

We designed a survey to measure levels of experience, confidence and knowledge of tic disorders among HCPs working in CYP’s mental health, paediatric and neurology services in England. The survey was developed with advice from the national charity Tourettes Action and healthcare experts (including coauthors TM, JK, NJ).

#### Participants

HCPs including psychiatrists, psychologists, paediatricians, neurologists and mental health nurses working in healthcare services for CYP in England were invited to participate via email. The study advert was circulated to these professionals by professional bodies such as the British Association of Neurologists and British Psychological Society and charities, such as Tourettes Action and ADHD Foundation-UK. HCPs who completed the survey were also asked to share it with their colleagues.

Hosted on Jisc V.2, the survey was live from December 2023 to early July 2024. Survey items included demographics such as region, job role, years working in the role and equality, diversity and inclusivity characteristics (table 1).

**Table 1:** National survey respondents’ demographic characteristics

Job role	% (n)	Gender	% (n)
Psychologist	27.2% (50)	Female	75.5% (139)
Paediatrician	24.5% (45)	Male	22.8% (42)
Mental health nurse	10.3% (19)	Prefer not to say	1.6% (3)
Psychiatrist	9.8% (18)	**Ethnicity**	**% (n**)
Paediatric neurologist	2.7% (5)	White British	71.7% (132)
Paediatric nurse	2.2% (4)	Other White	10.3% (19)
Speech and language therapist	1.6% (3)	Indian	3.8% (7)
Occupational therapist	1.6% (3)	White and Black	3.3% (6)
Assistant psychologist	1.6% (3)	African	2.7% (5)
Other	18.5% (34)	Chinese	1.1% (2)
**Years of experience**	**% (n**)	White and Black Caribbean	1.1% (2)
1–2	13.0% (24)	Other Asian	1.1% (2)
3–4	23.4% (43)	Other mixed or multiple	1.1% (2)
5–6+	63.6% (117)	Pakistani	0.5% (1)
**Years of experience assessing/treating CYP with tics**		Arab	0.5% (1)
<1	16.8% (31)	White and Asian	0.5% (1)
1–3	26.1% (48)	Prefer not to say	2.2% (4)
4–6	14.1% (26)	**Age group worked with**	**% (n**)
7–9	7.1% (13)	Under 5 years	44.6% (82)
10–12	10.9% (20)	5–11 years	92.4% (170)
13+	25.0% (46)	12–17 years	95.7% (176)
**Services worked in**	**% (n**)		
CAMHS	54.9% (101)		
Paediatric	34.2% (63)		
Neurology	6.5% (12)		
Private health	3.3% (6)		
Specialist tertiary	1.1% (2)		
Voluntary and charity sector	1.1% (2)		
Other	6.5% (12)		

CAMHS, Child and Adolescent Mental Health Services; CYP, children and young people.

The remaining survey questions used categorical scales to assess:

- Level of experiences of assessing and treating tics: ‘<1 year’; ‘1–3 years’; ‘4–6 years’; ‘7–9 years’; ‘10–12 years’ and ‘13 or more years’.
- Confidence in knowledge of tic disorders: ‘not at all confident’; ‘a little bit confident’; ‘somewhat confident’; ‘mostly confident’ and ‘extremely confident’.
- Awareness and use of published guidelines surrounding assessment and management of tics: response options included: ‘Yes, I am aware of them, and I use them’; ‘Yes, I am aware of them, but I do not use them’ and ‘No, I am not aware of any guidelines’.

Two open-ended items asked participants if they would like to provide any further information on their experiences of (1) assessing and (2) treating CYP with tic disorders.

##### Analysis

Descriptive statistics were reported (n and %). Free-text responses were organised by grouping similar comments into themes.

### Patient and public involvement

Members of the Tourette syndrome steering group, established at the University of Nottingham, with lived experience of tic disorders were involved in research question and design development. EMcN, a member of the study team and a parent with lived experience, provided input on devising FOI and national survey questions and assisted with survey recruitment and sending FOI requests. Results were disseminated to study participants via an online seminar event. Findings were also shared with the INTEND PPI panel, consisting of 10 parents/carers of CYP with tics, who reflected on them based on their lived experience. Following study completion, the study team planned to co-produce dissemination materials with the PPI panel.

## Findings

### Freedom of information requests

#### Freedom of information first request

Of 42 ICBs contacted, 34 ICBs responded, covering 234 places, with the remaining ICBs stating that they did not hold the requested data (n=7) or that the requested data were exempt from the Freedom of Information Act (n=1). Of the 34 responses gathered, one ICB did not provide information for all their places and one ICB only answered some questions. Responses were analysed for 234 places across 34 ICBs.

CYP with tics were most commonly referred to paediatric services (n=91/234 places, 38.9%), followed by Child and Adolescent Mental Health Services (CAMHS, n=77/234 places, 33%). However, 36 places (15.4%) stated that referrals were only accepted if the primary reason for the referral was a mental health or neurodevelopmental condition meeting the threshold for referral to the service; tics were not considered sufficient to accept the referral. Responses also indicated that CYP were signposted to charities and support groups and accessed support through community services and third sector organisations.

Of 234 places, 166 (70.9%) responded to the question about plans to improve tic services, with most responding that they had no current plans (n=71/166, 42.8%), compared with 56/166 places (33.7%) stating that they did have plans to improve. Of the places that responded to the question about interest in training for clinical staff (n=150, 64.1%), many showed interest in receiving training (n=89/150, 59.3%). Of places that responded to the question about whether they would like to receive information about a recommended service pathway (n=151, 64.5%), most were interested (n=117, 77.5%).

#### Freedom of information second request

Eighty services had been named by ICBs in FOI1 as providers of tic services for CYP. FOI2 was sent to these 80 contacts to gather data on the numbers of CYP with tics accepted into the service, assessed and treated. Sixty-two responses were received (77.5% of 80 contacted). The responses revealed that not all include tic disorders within their service provision, despite having been named as service providers in FOI1. We categorised these 80 responses as: services that provide a full pathway for tics (n=12, 15%); a partial pathway (n=9, 11.3%); no pathway (n=35, 28%); information not available (n=13, 16.3%) and did not respond (n=18, 22.5%). Full pathways were defined as those offering both assessment and treatment of tics. Partial pathways accepted referrals for CYP with tics (irrespective of comorbid conditions) and conducted diagnostic assessment, but did not offer treatment. Providers categorised as no pathway only assessed tics when the referral was for another neurodevelopmental or mental health assessment or declined referrals and/or signposted patients elsewhere.

**Figure 1:**
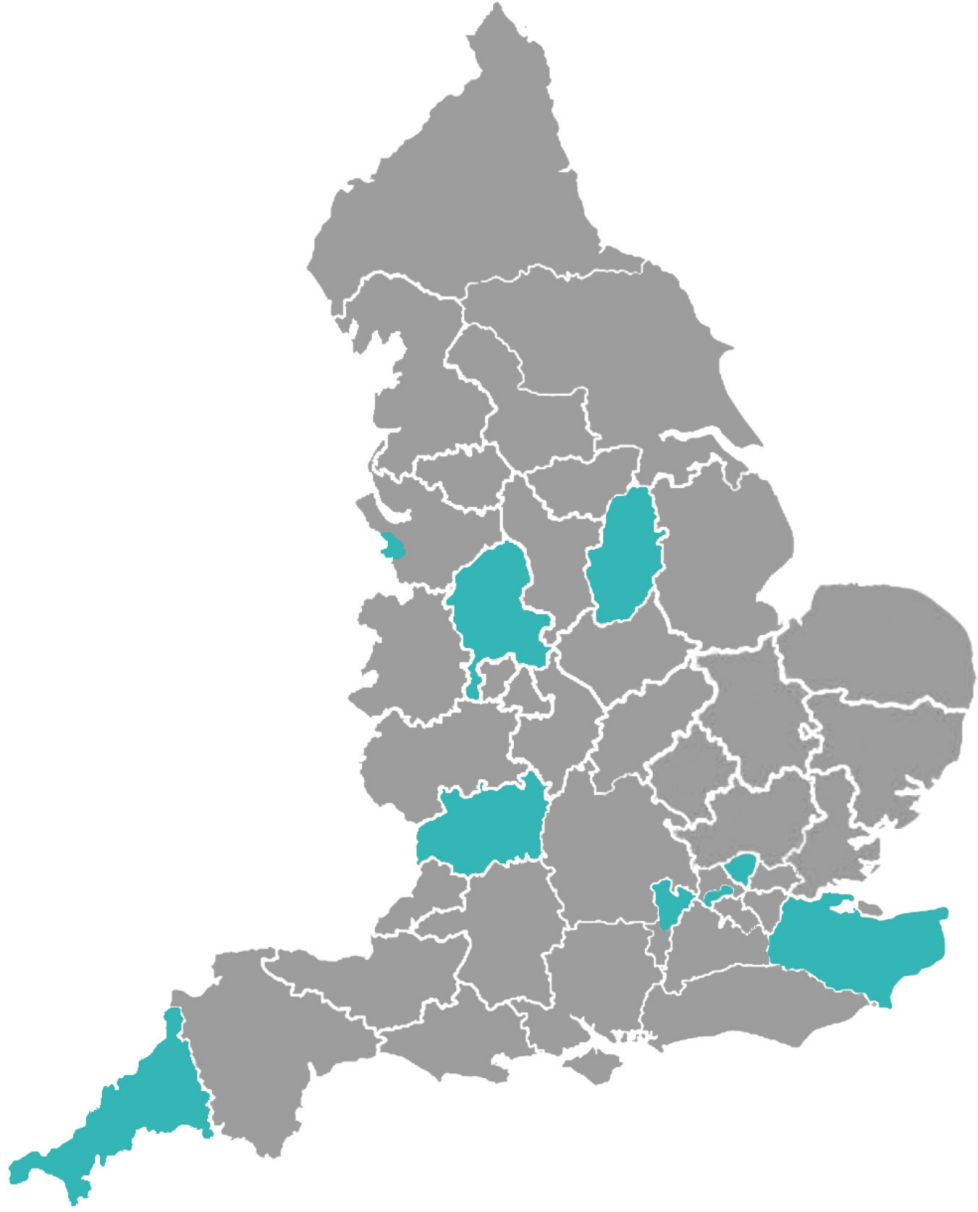
Integrated Care Board map displaying geographical regions where full pathway providers are located in England.

Full pathway providers were mapped based on their geographical location (figure 1). Service providers offering a full pathway were concentrated in the region of London, followed by the South-West and East Midlands, with minimal provision in the West Midlands, South-East and North-West. Notably, there were no full pathway providers in the upper North or East of England.

Of 12 providers with a full pathway, seven provided some data (table 2) for the number of referrals of tic disorders received and accepted, and the number of CYP assessed and treated. However, providers reported that data were often not recorded or not in a reportable format (table 2). Providers varied in the types of treatment offered, with medication and behavioural therapy more commonly offered than family interventions.

**Table 2:** FOI2 data on referrals received and accepted and number of CYP assessed and treated*

Type of provider	Referrals received	Referrals accepted	Diagnostic assessment	Family intervention	Medication	Behavioural therapy	Psycho-education
CMH trust 1	NR	160	NR	NR	NR	NR	160
CMH trust 2	131	78	NR	DA	143	NR	NR
Specialist trust 1	29	29	29	NO	15	NO	NO
MH trust 1	334	319	130	DA	NR	39	NR
Acute trust 1	10–15	10–15	NR	NO	<5	5	10
MH trust 2	NR	8	<5	NO	<5	<5	<5
Specialist trust 2	200	114	136	NO	NO	36	148

* Some CYP receiving assessment and treatment may have been on a waiting list prior to the data collection period and are therefore not included within the number of referrals accepted and received.

CMH, Community and Mental Health; CYP, children and young people; DA, declined to answer; FOI2, freedom of information second request; MH, mental health; NO, not offered; NR, not recorded.

### National survey of healthcare professionals

A total of 198 participants completed at least part of the survey. Fourteen participants were excluded: four were not HCPs and 10 did not continue beyond the initial demographics section. The final sample for analysis consisted of 184 HCPs.

Participants worked in a range of clinical services seeing CYP from various age groups (table 1). All HCPs in the sample stated that they saw CYP with tics (n=184, 100%) but varied in their years of experience assessing and treating tics. Less than half reported being aware of and using clinical guidelines for tics (table 3), with HCPs being most commonly aware of and using European (n=35, 19.0%) and NICE (n=28, 15.2%) guidelines.

**Table 3:** Healthcare professionals’ experience in assessing and treating tics

		% (n)
**Awareness and use of guidelines for tics**		
Aware and use them		49.5% (91)
Aware, but do not use		9.8% (18)
Not aware		40.8% (75)
	**% (n**)	**% (n**)
**Training**		
	**Assessment**	**Treatment**
Yes	46.2% (85)	50.0% (92)
No	53.8% (99)	50.0% (92)
**Confidence**		
	**Assessment**	**Treatment**
Extremely	9.2% (17)	5.4% (10)
Mostly	36.4% (67)	31.0% (57)
Somewhat	25.5% (47)	20.7% (38)
A little bit	13.0% (24)	17.9% (33)
Not at all	6.5% (12)	14.7% (27)
N/A (do not assess/treat)	9.2% (17)	10.3% (19)
**Need for further resources**		
	**Assessment**	**Treatment**
Yes	81.5% (150)	79.3% (146)
No	5.4% (10)	5.4% (10)
Maybe	13.0% (24)	15.2% (28)

#### Assessing tics

Most HCPs (n=131, 71.2%) reported conducting assessments of tic disorders in CYP but over half had not received training in assessment (table 3). HCPs typically reported feeling ‘mostly’ confident (n=67, 36.4%) when carrying out tic assessments and used a variety of tools for assessing tics, including clinical interviews (n=133, 72.3%) and the Yale Global Tic Severity Scale (n=71, 38.6%) (YGTSS).^19^

##### Treating tics

Most respondents reported providing treatment for CYP with tics (n=127, 69.0%). Half had received training in treating tics (n=92, 50.0%). Confidence ratings indicated lower confidence when treating compared with assessing tics.

Some HCPs prescribed medication, with clonidine (n=35, 19.0%), aripiprazole (n=25, 13.6%) and guanfacine (n=22, 12.0%) being the most frequently prescribed. However, more HCPs used cognitive behavioural treatments, such as habit reversal training (HRT) (n=81, 44.0%) and ERP (n=66, 35.9%).

Most HCPs stated ‘yes’ to needing further resources and training for assessing (n=150, 81.5%) and treating tics (n=146, 79.3%).

##### Qualitative analysis of free-text survey responses

Free-text responses to the survey items provided further information, organised thematically as follows.

##### Constraints of assessments

HCPs often identified and assessed tics when completing wider mental health or neurodevelopmental assessments. However, in these cases, tics were commonly only considered as a co-occurring condition, with comorbidities also making it more difficult to assess tics and their impact. Services were under-resourced to meet clinical demands and therefore assessments could not sufficiently include evaluation of comorbidities and distress. While assessment tools were useful, some HCPs favoured the development and use of a single, holistic, but efficient assessment. Respondents also highlighted the need for training in how to use and score assessment tools effectively, and how to diagnose complex, comorbid presentations.

##### Difficulties with locating available treatment

Some HCPs commented that tic assessments felt futile when there was often no treatment available. HCPs were frustrated by the inadequacy of care pathways, and some described having difficulty liaising with other services, with referrals for tics often rejected, even when clearly within the service’s remit.

##### Resource and financial restrictions

HCPs stated that many services were not commissioned to manage tics and where they were, funding was unstable and thus service provision fluctuated. One respondent described commissioning for CAMHS clinicians to be ‘rigid’, not allowing them to engage with patients with tics. Others also felt that there were no clear guidelines and often no training around tics within their service. This suggests that, although CAMHS is often considered the responsible service, it may frequently be ill-equipped to manage tics effectively.

##### Issues with service structure/organisation and suggested improvements

Some HCPs commented that services that managed tics were often inadequate, with some offering assessment but no treatment, or providing treatment but not assessing tics. In some instances, there was no service to refer into for treatment, which aligns with the findings from the FOI requests above. Furthermore, respondents noted a lack of multidisciplinary input, with some HCPs feeling that their service was missing psychological input, while others felt that medical and psychiatric input was absent.

Some described offering medication due to a lack of alternative treatment options, despite behavioural therapies being the recommended treatment in published guidelines. Many HCPs stated that they would like NICE guidelines for the assessment and treatment of tic disorders as they currently consult other resources, such as training manuals and European guidelines.^12 20 21^ Many also felt that there should be capacity to deliver alternatives to medication, desiring training and more support with delivering first-line psychological therapies.

A lack of formal training was suggested to at least partially contribute to lower confidence when assessing tics. HCPs’ knowledge around tics seemed to instead develop through clinical practice and working with highly experienced clinicians where available.

## Discussion

This research assessed how services for tic disorders are currently organised in England, using FOI requests. Experiences and training needs of HCPs were captured via a national survey.

### Freedom of information requests

FOI requests revealed that service provision for CYP with tics across England was very limited. Available services were typically provided through paediatric teams and CAMHS, which is appropriate given the co-occurrence of tic disorders with other mental health and neurodevelopmental difficulties. However, most of these service providers only accept referrals if there is another mental health or neurodevelopmental need meeting their referral thresholds. Tics were rarely a sufficient referral criterion on their own, regardless of the degree of functional impairment or harm caused by the tics, or their chronicity. Fewer than 20% of providers who responded to FOI requests offered a full care pathway including referral, assessment and treatment for tics, highlighting the fragmentation of service provision and barriers to accessing care.^9 10 14 22^ Services also reported signposting to third sector organisations including charities and support groups (consistent with approaches for other neurodevelopmental disorders). These provide valuable support but cannot offer diagnostic assessment or treatment. Furthermore, there were regional inequalities in access to these full pathways. Of the 12 providers with a full care pathway, five were concentrated in London, with none in the upper North-East and East of England.

Quantifying the impact of inconsistent tic service provision was challenging, as many places did not record the number of tic referrals received or accepted. Some providers cited the absence of a dedicated Systematised Nomenclature of Medicine Clinical Terms (SNOMED CT)^23^ code for tic disorders as the reason. However, specific SNOMED codes for tic disorders do exist (SNOMED CT ID: 386783003), suggesting that service providers need better knowledge or support around including them in electronic health records. In addition, some ICBs were unaware of how tic services functioned locally, indicating poor communication between services.

### National survey of healthcare professionals

While over 70% of 184 respondents in the national survey sample reported assessing tics, with the YGTSS and clinical interviews being the most commonly used tools, less than half the sample had received training in these assessment methods. Despite a notable proportion of the sample (~40%) being unaware of guidelines for tics and having not received training, a high proportion of respondents reported feeling mostly confident when carrying out tic assessments. However, this was followed by a considerable proportion, around one in four, feeling only somewhat confident.

Similarly, nearly 70% of the sample reported that they treat tics, with behavioural treatments such as HRT and ERP being the most common methods. However, only half reported receiving training. Confidence was also lower when treating compared with assessing tics. Overall, this suggests that HCPs provide healthcare to CYP with tics, despite not always feeling confident or being trained.

Barriers to treating and assessing tics included a lack of available resources, such as funding and guidelines, coupled with incomplete care pathways. This was accompanied by a lack of training, which impacted clinicians’ confidence. Some noted that current NICE guidelines were not sufficiently informative, not specific to the English healthcare system and did not directly pertain to tic disorders, but rather neurological conditions.^24^

HCPs reported being unsure which local service was responsible for managing tics, often feeling exasperated by patients’ referrals being rejected and passed between services without progress. Appropriate commissioning is required to ensure greater clarity and cohesiveness in tic service provision and to ensure ICBs are providing healthcare to meet the needs of the local population.^25^ Evidence suggests this may also lead to cost savings.^11^

### Strengths and limitations

The FOI requests provided an understanding of the current climate of tic services, including regional differences in availability. This has previously not been explored in depth in England and provides context to existing patient accounts that tic services are inadequate.^10 22^ Furthermore, since the FOI data came from institutional records, and NHS organisations are required to respond to reasonable requests, this method reduces the bias that may arise from self-reported data or survey samples and increases the representativeness of the responses received. However, FOI requests as a research method also have some shortfalls. For example, some contacts did not respond despite their legal obligation to do so, and some responses provided inaccurate information surrounding tic service providers. Many responses also lacked detail, leading to a need to contact respondents again for clarification. It is also possible that high-performing services were more likely to respond to the FOI request, potentially leading to a positive bias in the pattern of findings.

The national survey sample was diverse, both in terms of job role and years of experience. Furthermore, all age groups of CYP were seen by clinicians within the sample. However, the national survey sample consisted primarily of female and White British HCPs. This may be representative of the HCP population in England,^26 27^ but further research targeting a broader range of professionals is needed. Additionally, while HCPs in the sample worked in a variety of services, few were from specialist services, meaning that the findings may have lacked specialist insight, including positive experiences within specialist tertiary services. In addition, the sample size may reduce the generalisability of the findings, although the consistent pattern of responses and representation from all main groups of HCPs likely to work in services receiving referrals for tics suggests findings are representative of these main groups. Finally, it is possible that respondents were primarily those who are interested in tics, and the survey findings will therefore reflect views from professionals with these backgrounds.

### Clinical implications

Without cohesive services and trained professionals, diagnosis and treatment are likely to be delayed,^10^ which may increase tic severity^28^ and contribute to poorer quality of life and psychosocial functioning^29^ and negative long-term outcomes, such as increased risk of suicide.^4^ Without clear pathways, resources may be used inefficiently, as CYP are referred to multiple services—resulting in greater cost to the NHS.^11^ CYP with tic disorders should be able to access specialist care within existing mental health and neurodevelopmental services when this is clinically indicated, regardless of whether there are other co-occurring conditions also requiring assessment and treatment. There should be a clear route from referral to diagnostic assessment and, where needed, evidence-based treatment. Where co-occurring conditions exist, these should be assessed and managed in addition to the tics, to support holistic, needs-led care for each child or young person. Exact specifications of how this is managed are best determined by local ICBs/service providers.

More broadly, FOI findings and HCPs’ responses show that the current organisation of tic services does not align with the NHS Long Term Plan,^30^ which strives to reduce healthcare disparities, improve healthcare quality, ensure HCPs have sufficient training and ensure the appropriate allocation of NHS budget. Our findings highlight the pressing requirement to allocate more funding to tic service provision across England and to develop more effective training for HCPs, including knowledge of existing guidelines. Furthermore, although the lack of specialised training and access to appropriate resources affects the provision of neurodevelopmental services more widely, analysis of the free-text responses gathered from the HCP survey indicates that referrals are rejected because services are not commissioned to manage tics. The poor reporting of tic referrals, assessment and treatment uncovered in our FOI requests suggests that the prevalence of tic disorders in CYP in England is likely to be under-reported. This may directly contribute to tics in CYP being de-prioritised as an area of improvement in healthcare service delivery by ICBs. Further research should investigate the true prevalence of tics in CYP to ensure adequate funding and service provision to meet the needs of this population.

### Conclusion

The current research highlighted inconsistent, limited tic service provision for CYP across England. HCPs report a need for more training, clearer clinical guidelines and described insufficient funding of tic services. Most places in FOI1 had no immediate plans for improvement but were interested in clinical training and receiving information about a best practice model. There is an urgent need to enhance access to assessment and treatment for CYP with tics and improve training and resources for HCPs.

## Data Availability

Data are available on reasonable request.

## References

[1] Ueda K, Black KJ (2021). A Comprehensive Review of Tic Disorders in Children. J Clin Med.

[2] Małek A (2022). Pain in Tourette Syndrome-Children’s and Parents’ Perspectives. J Clin Med.

[3] Eapen V, Cavanna AE, Robertson MM (2016). Comorbidities, Social Impact, and Quality of Life in Tourette Syndrome. Front Psychiatry.

[4] Fernández de la Cruz L, Rydell M, Runeson B (2017). Suicide in Tourette’s and Chronic Tic Disorders. Biol Psychiatry.

[5] Evans J, Seri S, Cavanna AE (2016). The effects of Gilles de la Tourette syndrome and other chronic tic disorders on quality of life across the lifespan: a systematic review. Eur Child Adolesc Psychiatry.

[6] Pérez-Vigil A, Fernández de la Cruz L, Brander G (2018). Association of Tourette Syndrome and Chronic Tic Disorders With Objective Indicators of Educational Attainment: A Population-Based Sibling Comparison Study. JAMA Neurol.

[7] Malli MA, Forrester-Jones R (2022). Stigma and Adults with Tourette’s Syndrome: “Never Laugh at Other People’s Disabilities, Unless they have Tourette’s—Because How Can You Not?”. J Dev Phys Disabil.

[8] Two technologies recommended for people with chronic tic disorders and Tourette syndrome (2024). National institute for health and care excellence. https://www.nice.org.uk/news/articles/two-technologies-recommended-for-people-with-chronic-tic-disorders-and-tourette-syndrome.

[9] Hollis C, Pennant M, Cuenca J (2016). Clinical effectiveness and patient perspectives of different treatment strategies for tics in children and adolescents with Tourette syndrome: a systematic review and qualitative analysis. Health Technol Assess.

[10] Marino C, Khan K, Groom MJ (2023). Patients’ experience of accessing support for tics from primary care in the UK: an online mixed-methods survey. BMC Health Serv Res.

[11] Hall CL, Le Novere M, Murphy T (2024). Healthcare utilisation and costs associated with poor access to diagnosis and treatment for children and young people with tic disorders. BMJ Ment Health.

[12] Andrén P, Jakubovski E, Murphy TL (2022). European clinical guidelines for Tourette syndrome and other tic disorders-version 2.0. Part II: psychological interventions. Eur Child Adolesc Psychiatry.

[13] Whittington C, Pennant M, Kendall T (2016). Practitioner Review: Treatments for Tourette syndrome in children and young people - a systematic review. J Child Psychol Psychiatry.

[14] Cuenca J, Glazebrook C, Kendall T (2015). Perceptions of treatment for tics among young people with Tourette syndrome and their parents: a mixed methods study. BMC Psychiatry.

[15] Perkins V, Coulson NS, Davies EB (2020). Using Online Support Communities for Tourette Syndrome and Tic Disorders: Online Survey of Users’ Experiences. J Med Internet Res.

[16] Bhikram T, Elmaghraby R, Abi-Jaoude E (2021). An International Survey of Health Care Services Available to Patients With Tourette Syndrome. Front Psychiatry.

[17] Parker A, French B, Groom MJ (2024). Systematic review-understanding the barriers and facilitators experienced by healthcare professionals in providing care for tics: a mixed methods systematic review of clinical knowledge, attitudes, and practices. BMC Med Educ.

[18] Clifton-Sprigg J, James J, Vujić S (2020). Freedom of Information (FOI) as a data collection tool for social scientists. PLoS One.

[19] Leckman JF, Riddle MA, Hardin MT (1989). The Yale Global Tic Severity Scale: initial testing of a clinician-rated scale of tic severity. J Am Acad Child Adolesc Psychiatry.

[20] Szejko N, Robinson S, Hartmann A (2022). European clinical guidelines for Tourette syndrome and other tic disorders-version 2.0. Part I: assessment. Eur Child Adolesc Psychiatry.

[21] Roessner V, Eichele H, Stern JS (2022). European clinical guidelines for Tourette syndrome and other tic disorders-version 2.0. Part III: pharmacological treatment. Eur Child Adolesc Psychiatry.

[22] Ludlow AK, Brown R, Schulz J (2018). A qualitative exploration of the daily experiences and challenges faced by parents and caregivers of children with Tourette’s syndrome. J Health Psychol.

[23] (2025). NHS england » snomed ct. https://www.england.nhs.uk/digitaltechnology/digital-primary-care/snomed-ct/.

[24] National Institute for Health and Care Excellence (2019). Suspected neurological conditions: recognition and referral. https://www.nice.org.uk/guidance/ng127.

[25] Independent investigation of the NHS in England (2024). Department of Health and Social Care. GOV.UK.. https://www.gov.uk/government/publications/independent-investigation-of-the-nhs-in-england.

[26] Workforce Census 2022 - Full report (2022). Royal college of paediatrics and child health. https://www.rcpch.ac.uk/resources/workforce-census-2022-full-report.

[27] NHS Workforce Statistics - August 2024 (Including selected provisional statistics for September 2024) (2025). NHS England Digital.

[28] Iverson AM, Black KJ (2022). Why Tic Severity Changes from Then to Now and from Here to There. J Clin Med.

[29] O’Hare D, Helmes E, Eapen V (2016). The Impact of Tic Severity, Comorbidity and Peer Attachment on Quality of Life Outcomes and Functioning in Tourette’s Syndrome: Parental Perspectives. Child Psychiatry Hum Dev.

[30] (2025). NHS long term plan. https://www.longtermplan.nhs.uk/.

